# Sex inequality in under-five deaths and associated factors in low and middle-income countries: a Fairlie decomposition analysis

**DOI:** 10.1186/s12889-022-12679-y

**Published:** 2022-02-16

**Authors:** Adeniyi Francis Fagbamigbe, Oyewale Mayowa Morakinyo, Folusho Mubowale Balogun

**Affiliations:** 1 Department of Epidemiology and Medical Statistics, Faculty of Public Health, College of Medicine, University of Ibadan, Ibadan, Nigeria; 2 Department of Environmental Health Sciences, Faculty of Public Health, College of Medicine, University of Ibadan, Ibadan, Nigeria; 3 Institute of Child Health, College of Medicine, University of Ibadan, Ibadan, Nigeria

**Keywords:** Sex inequality, Under-five death, Fairlie decomposition, Low- and middle-income countries

## Abstract

**Background:**

There exist sex disparities in the burden of Under-five deaths (U5D) with a higher prevalence among male children. Factors explaining this inequality remain unexplored in Low-and Medium-Income Countries (LMIC). This study quantified the contributions of the individual- and neighborhood-level factors to sex inequalities in U5D in LMIC.

**Methods:**

Demographic and Health Survey datasets (2010-2018) of 856,987 under-five children nested in 66,495 neighborhoods across 59 LMIC were analyzed. The outcome variable was U5D. The main group variable was the sex of the child while individual-level and neighborhood-level factors were the explanatory variables. Fairlie decomposition analysis was used to quantify the contributions of explanatory factors to the male-female inequalities in U5D at *p*<0.05.

**Results:**

Overall weighted prevalence of U5D was 51/1000 children, 55 among males and 48 among females (*p*<0.001). Higher prevalence of U5D was recorded among male children in all countries except Liberia, Kyrgyz Republic, Bangladesh, Nepal, Armenia, Turkey and Papua New Guinea. Pro-female inequality was however not significant in any country. Of the 59 countries, 25 had statistically significant pro-male inequality. Different factors contributed to the sex inequality in U5D in different countries including birth order, birth weight, birth interval and multiple births.

**Conclusions:**

There were sex inequalities in the U5D in LMIC with prominent pro-male-inequality in many countries. Interventions targeted towards the improvement of the health system that will, in turn, prevent preterm delivery and improve management of prematurity and early childhood infection (which are selective threats to the male child survival) are urgently required to address this inequality.

## Background

The under-five death (U5D) rate is the probability of a child dying before attaining five years of age. It remains the most useful indicator of child wellbeing [1] which reflects the overall strength of the health system of countries and the value the society place on health care [2]. The Millennium Development Goal (MDG) 4 sought to reduce the global under-five mortality rate by two-thirds between 1990 and 2015. Concerted efforts were made to ensure the realization of this goal through the promotion of exclusive breastfeeding, good nutrition, optimal vaccinations, correct management of common childhood infections, ensuring a safe environment (through improved sanitation and prevention of pollution) and access to potable water [3]. These interventions led to the prevention of about 4 million U5D between 2000 and 2015 [4]. The reduction was most dramatic in countries with the highest U5D [3]. However, the majority of U5D recorded at this time was from sub-Saharan Africa (almost half of the cases) and Southern Asia (a third of the cases) [3, 4] and these were mainly low-and middle-income countries (LMIC). It was therefore not surprising that most of these countries could not meet up with the MDG 4 goal.

There were inequalities in the reported U5D within LMIC with variations across location, sex and socio-economic class [5]. Sex differences in U5D have been observed in different parts of the world and the pattern depends on the socio-economic development of countries. For developing countries, the natural setting supports female survival advantage [6]. This is because male infants have some inherent biological features which make them less likely to survive compared to females. These features include the effect of the x-linked immunoregulatory genes which gives females more resistance to infection [7, 8]. Waldron affirmed that higher proportions of males being born prematurely with the accompanying complications and there is the  risk of lung immaturity in males as a result of the effect of testosterone on the lungs which predispose them to respiratory distress syndrome [7]. For developed countries, male disadvantage disappeared over the years as a result of the improvement in child healthcare [6, 9] but the female survival advantage persists till age five [10]. However, the natural female advantage for survival can be lost in settings where females are deprived of access to health care [11] and good nutrition, as well as exposure to harmful environments [6, 9]. This is a common finding in many LMIC where male preference have persisted as a result of deep seated patriarchal cultures seen in these countries [12].

It is not clear if the earlier listed interventions had benefitted both male and female children equally or if there is a sex disparity in their survival. In the presence of disparity, it will be imperative to investigate the factors which contribute to sex disparity in U5D as this can reveal specific contributions to U5D and provide guidance for more targeted approaches and intervention in addressing the problem. A cursory examination of most national U5D  data will mask inequalities and not make them obvious. This can hinder the invention of novel and equally effective approaches that can work synergistically with existing strategies to effectively address U5D . The United Nations suggested that a closer look at existing data can give rise to more novel approaches to flatten the curve of U5D in developing countries [3]. One way of achieving this is the decomposition of factors that contribute to the disparity. This will allow for the appreciation of more intricacies in the factors that could be associated with the inequality and its outcomes can be an invaluable tool that can lead to the production of cut-edge interventions to reverse the trend of U5D in developing countries. More efforts are required in LMIC where the bulk of U5D still occur for these regions to achieve the goal of Sustainable Development Goal (SDG) 3.2, which is to ensure health for all ages [13]. Also, the countries which succeeded in achieving the MDG 4 need to keep up the efforts to achieve SDG 3.2 by continuing the effective interventions and exploring other novel approaches . Therefore, the goal of this study is to decompose the factors associated with sex differences in U5D in the LMIC. We posit that due to the male preference culture that is prevalent in many LMIC, there will likely be more pro-female U5D inequality in these countries.

## Methods

### Study design and data

We obtained under-five children data from the Demographic and Health Surveys (DHS). The DHS holds approximately every five years across the participating LMIC. The ICF (USA) collects the data in conjunction with the designated organizations in the participating countries. Typically, the survey is cross-sectional, nationally representative and population-based. We pooled data from the most recent DHS conducted between 2010 and 2018 and in the public domain as of 10 September, 2020. A total of 59 LMIC met these inclusion criteria, and their data were included in this study. We pooled the data of 856,987 under-five children, from 66,495 neighborhoods across the 59 LMIC.

### Sampling strategies

The DHS utilized a similar clustered multi-stage sampling procedure in the participating countries based on countries’ sampling frames drawn mostly from the last census counts. Countries were stratified using the existing geographical and administrative structures. The multi-stage mechanism included the states/divisions/regions in the first stage, districts as the next stage in some countries, and finally, the clusters as the last stage. The clusters were the primary sampling units (PSU) [14, 15]. The households were then selected from the PSUs, from which women aged 15-49 years were interviewed.

The surveys generated different datasets. We used the child recode data that captured diverse information on all births of the interviewed women five years before the survey. Sampling weights were computed and provided alongside the data by DHS. These computations were based on the multi-stage sampling method to ensure the representativeness of the sample concerning the general population. The DHS uses similar surveys and research protocols, standardized questionnaire, similar interviewer training, supervision, and implementation in all the countries. The full details of the sampling methodologies and other information are available at dhsprogram.com.

### Variables

#### Dependent variable

The dependent variable was UD5 which was defined as death among live births within the first five years of life, that is deaths within 0 to 59 months of birth [14]. To ascertain the correctness of this outcome, mothers were first asked if they had given birth to any child five years preceding the date of the study. They were then asked to recount the date of birth and were assisted to estimate such dates when necessary. They were asked if each of those children were alive or dead. The dates of death or the ages at death for the dead children were then used to determine U5D. Therefore, U5D was binary: Alive or Dead before 5^th^ birthday.

#### Main group variable

The main group variable is the sex of the child: male or female.

#### Independent variables

The variables identified to be associated with childhood deaths in the literature [16–20] were selected using Moseley’s systematic conceptual framework on the study of child survival in developing countries [17]. The variables were made up of individual-level and neighborhood-level factors.

##### Individual-level factors

The individual-level factors consist of a child’s characteristics, mothers’ characteristics and the households’ characteristics. Child's characteristics were weight at birth (average+, small and very small), birth interval (firstborn, <36 months and >=36 months). birth order (1, 2, 3 and 4+) and whether a child is a twin (single, multiple (2+)). While mothers’ characteristics were maternal education (none, primary or secondary plus), maternal age (15 to 24, 25 to 34, 35 to 49 years), marital status (never married, currently and formerly married), maternal and paternal employment status (working or not working), and health insurance (yes or no). Households’ characteristics were the sex of the head of the household (male or female), access to media (at least one of radio, television or newspaper), sources of drinking water (improved or unimproved), toilet type (improved or unimproved), cooking fuel (clean fuel or biomass), housing materials (improved or unimproved), household wealth index (poorest, poorer, middle, richer and richest) and place of residence (rural or urban). The sources of clean fuel are electricity, liquefied natural gas/biogas and unclean fuel include wood, charcoal, kerosene, straw shrubs, animal dungs and grass. The improved sources of drinking water include a protected well, borehole, bottled water and spring rainwater, while spring water, tankers, unprotected well with drum, sachet water, surface water, and other sources constituted the unimproved sources. The housing material was based on a composite score according to the type of wall, floor and roof materials. If cement/carpet/rug/ceramic tiles/vinyl asphalt strips were used for the floor, the floor quality is coded 1, else it is coded 0. In the same vein, wall material quality is coded 1 if it is made of cement blocks/bricks, else 0. If roof material is made of calamine/cement roofing shingles/cement fibres/ceramic tiles/zinc, it is coded 1, else 0. If all the materials fall under “1” they are regarded as “improved”, else, they are “unimproved” [14, 15].

##### Neighborhood-level factors

We defined “neighborhood” as the clustering of children. The DHS uses “clusters” as the PSU [14, 15], hence “neighborhood” in this context is the clustering of children within the same geographical environment and children were “neighbors” if they belonged to the same cluster. In this study, we considered neighborhood socioeconomic status (SES) as a neighborhood-level variable. It was computed using the principal component factor method from the scores that were aggregated from the proportion of respondents within the same clusters without education, belonging to a household in the two lowest wealth quintiles, no media access and unemployed. The “xtile” function in Stata version 16 was used to categorize the scores into five categories: Least disadvantaged, 2, 3, 4 and most disadvantaged [5].

### Statistical analyses

We used both descriptive and inferential statistics in this study. Descriptive statistics including charts, tables, and percentages were used to show the distribution of the children by country, regions, U5D and other key variables. A bivariable analysis was conducted using the Z-test for equality of proportions of U5D among male and female children within each country and region (Table 1). We also determined if any association existed between the explanatory variables and U5D among the male and female children (Table 2). The risk difference (RD) in under-5 deaths among male and female children were computed. A RD greater than 0 suggests that U5D is higher among male children than among female children (pro-male inequality). While a RD = 0 signifies no difference and a negative RD indicates that U5D was higher among female children than among male children (pro-female inequality). The RDs were computed to identify the countries where significant differences existed in the U5MR by gender.

**Table 1:** Distribution of sample characteristics and prevalence of under-five deaths in LMIC by sex, 2010–2018

Country	No of Children	Survey year	No of Communities	Male (%)	Under-5 Death per 1000		
					Overall U5D	Male U5D	Female U5D
Overall	856,987		66495	51.4	51	55	48^a^
Eastern Africa	109,945		6298	50.7	52	57	47^a^
Burundi	13,192	2011	554	50.5	59	62	55
Comoros	3,149	2012	252	50.7	42	44	40
Ethiopia	10,641	2016	643	51.9	55	67	42^a^
Kenya	20,964	2014	1593	50.8	44	46	42
Malawi	17,286	2016	850	50.1	49	55	42^a^
Mozambique	11,102	2011	610	50.6	74	79	70
Rwanda	7,856	2014	492	50.4	39	43	34^a^
Tanzania	10,233	2015	608	50.7	53	57	48^a^
Uganda	15,522	2016	696	50.4	51	58	44^a^
Middle Africa	76,790		2932	50.5	70	77	64^a^
Angola	14,322	2016	625	49.8	51	58	45^a^
Cameroon	9,733	2018	429	51.5	62	67	56^a^
Chad	18,623	2015	624	51.2	98	108	88^a^
Congo	9,329	2012	384	49.6	51	57	46^a^
Congo DR	18,716	2014	536	49.7	75	77	72
Gabon	6,067	2012	334	51.8	53	63	43^a^
Northern Africa	15,848		876	52.7	24	26	22
Egypt	15,848	2014	876	52.7	24	26	22
Southern Africa	27,823		2549	49.9	51	55	46^a^
Lesotho	3,138	2014	397	49.5	69	70	69
Namibia	5,046	2013	537	48.8	45	46	44
South Africa	3,548	2016	671	52.1	36	42	30
Zambia	9,959	2018	545	50.2	49	56	42^a^
Zimbabwe	6,132	2015	399	49.1	57	63	50^a^
Western Africa	147,996		6099	50.8	81	86	75^a^
Benin	13,589	2018	555	51.0	70	78	62^a^
Burkina Faso	15,044	2010	573	50.7	89	94	85^a^
Cote d’Ivoire	7,776	2013	351	50.3	84	108	60^a^
Gambia	8,088	2013	281	50.7	41	41	40
Ghana	5,884	2014	427	52.2	46	50	43
Guinea	7,951	2018	401	51.9	87	89	83
Liberia	7,606	2013	322	51.1	70	70	70
Mali	9,940	2018	345	50.8	72	74	69
Niger	12,558	2012	476	50.5	81	84	77
Nigeria	33,924	2018	1389	50.9	97	100	93^a^
Senegal	6,719	2018	214	50.9	40	48	32^a^
Sierra Leone	11,938	2013	435	50.0	113	120	107^a^
Togo	6,979	2013	330	50.4	63	68	57
Central Asia	10,558		682	50.9	28	30	26
Kyrgyz Rep	4,363	2012	316	51.2	26	23	30
Tajikistan	6,195	2017	366	50.8	29	35	24^a^
South-Eastern Asia	17,716		1851	51.5	26	28	24
Cambodia	7,165	2014	609	50.1	29	31	26
Philippines	10,551	2017	1242	52.5	24	25	23
Southern Asia	338,925		33064	52.0	44	46	41^a^
Afghanistan	32,712	2015	956	51.6	47	49	45
Bangladesh	7,886	2014	600	52.1	41	39	43
India	259,627	2016	28332	52.2	44	47	41^a^
Indonesia	17,848	2017	1967	51.1	27	32	23^a^
Maldives	3,106	2016	265	50.9	18	21	15
Nepal	5,038	2016	383	52.3	34	32	37
Pakistan	12,708	2018	561	50.2	66	74	58^a^
Western Asia	28,475		2050	51.6	33	36	29^a^
Armenia	1,724	2016	306	53.3	5	4	6
Jordan	10,658	2017	964	51.6	17	19	16
Yemen	16,093	2013	780	51.4	45	50	40^a^
Central America	23,328		1996	52.1	28	30	26
Guatemala	12,440	2014	856	51.9	31	34	28
Honduras	10,888	2011	1140	52.4	25	26	24
South America	21,379		4788	50.7	16	17	15
Colombia	11,759	2015	3386	50.4	15	16	13
Peru	9,620	2012	1402	51.1	17	19	16
Southern Europe	6,410		688	52.1	10	09	10
Albania	2,762	2018	652	51.1	04	06	02
Turkey	3,648	2013	36	52.8	14	12	17
Caribbean	22,280		1863	51.5	47	51	42^a^
Dominican Rep	3,714	2013	518	51.7	29	33	25
Haiti	6,530	2016	450	50.3	69	74	64
Myanmar	4,815	2015	440	52.2	44	48	40
Timor Leste	7,221	2016	455	52.0	37	41	33
Oceania	9,514		759	52.5	40	40	40
Papua NG	9,514	2016	759	52.5	40	40	40
Total	856,987		66,495	51.4	51	55	48^a^

^a^Significant at 0.05 in the z-test of equality of proportions

**Table 2:** Characteristics of the studied children and prevalence of under-five-deaths in LMIC by sex, 2010–2018

Characteristics	n	%	Male %	Under-5 death per 1000		
				Overall	Male	Female
Maternal current age (years)						
15-24	254,644	29.7	51.5	53	58	48
25-34	442,799	51.7	51.5	47	50	44
35-49	159,544	18.6	50.9	61	64	57
Maternal highest educational						
No education	292,866	34.2	51.1	69	74	64
Primary	218,432	25.5	51	54	58	50
Secondary+	345,689	40.3	51.9	35	38	32
Media access						
No	340,783	40.5	50.9	66	70	61
Yes	500,111	59.5	51.7	43	47	40
Maternal employment						
Employed	324,757	53.3	50.8	61	65	57
Unemployed	284,531	46.7	51.3	45	49	40
Paternal employment						
Employed	541,347	95.8	51.1	55	60	51
Unemployed	23,796	4.2	51.5	48	53	44
Marital status						
Never married	27,341	3.2	50.6	52	57	48
Living With Sexual Partner	791,531	92.4	51.4	51	54	47
Formerly	38,110	4.4	50.9	63	67	59
Sex of household head						
Male	718,578	83.8	51.4	52	55	48
Female	138,409	16.2	51.3	51	54	47
Wealth index combined						
Poorest	221,239	25.8	50.8	62	67	57
Poorer	193,674	22.6	51.2	58	61	54
Middle	169,849	19.8	51.6	50	54	47
Richer	148,944	17.4	51.4	45	48	42
Richest	123,281	14.4	52.1	35	38	32
Covered by health insurance						
No	671,764	87.4	51.3	55	59	51
Yes	96,784	12.6	51.6	33	36	30
Child is twin						
Single birth	834,700	97.4	51.4	47	51	44
Multiple	22,287	2.6	50.5	198	216	18
Weight at birth						
Average+	686908	84.1	52.2	45	46	41
Small	94173	11.5	47.1	67	74	58
Very small	35624	4.4	46.3	116	132	101
Birth order						
1	243,300	28.4	51.8	48	53	43
2	205,906	24.0	51.5	41	44	37
3	138,761	16.2	51.7	46	48	45
4+	269,020	31.4	51.7	66	69	62
Birth interval						
1st Birth	243,305	28.5	51.8	48	53	43
<36 months	333,066	39.0	51.2	64	67	61
36+ months	278,326	32.6	51.2	38	41	35
Drinking water						
Unimproved sources	188,610	22.7	50.8	67	71	62
Improved source	641,485	77.3	51.5	47	51	44
Toilet type						
Unimproved sources	416,964	50.3	51.1	63	67	58
Improved source	412,803	49.7	51.6	40	43	37
Cooking fuel						
Unclean/biomass	620,900	76.6	51.0	60	64	56
Clean fuel	189,870	23.4	52.1	30	32	27
Housing material						
Unimproved material	500,644	62.7	51.0	61	65	56
Improved material	298,152	37.3	51.8	41	44	38
Place of residence						
Urban	235,866	29.5	51.5	42	44	39
Rural	562,930	70.5	51.2	58	59	51
Community SES Disadvantage						
Least disadvantaged	171,506	20.0	52.3	33	35	30
2	171,291	20.0	51.2	46	49	42
3	171,783	20.0	51.2	56	60	52
4	171,392	20.0	51.0	62	67	57
Most disadvantaged	171,015	20.0	51.2	62	66	58
Total	856,987	100.0	51.4	51	55	48

**Fig. 1:**
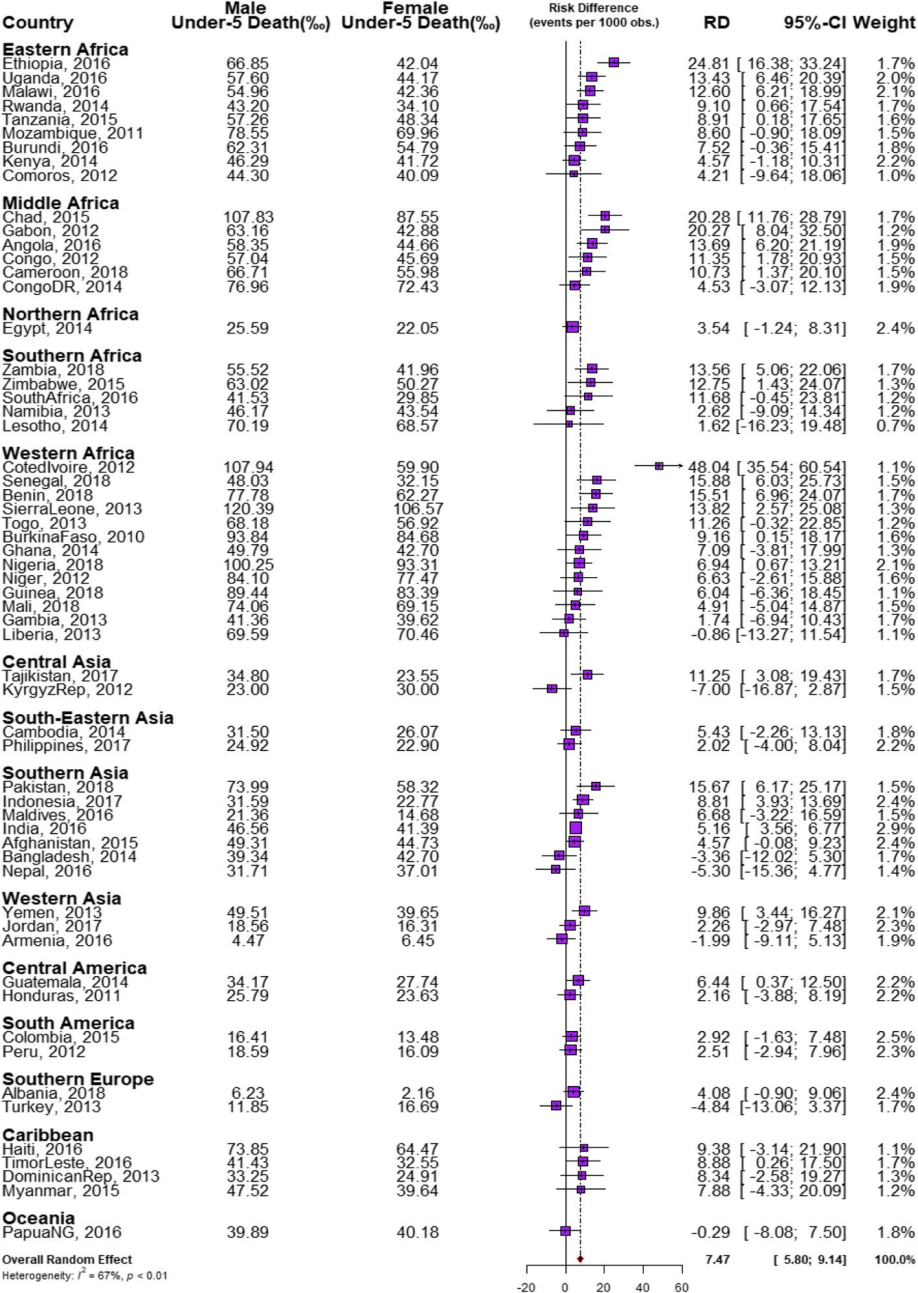
Risk difference in under-five deaths between male and female children by countries in LMIC

**Fig. 2:**
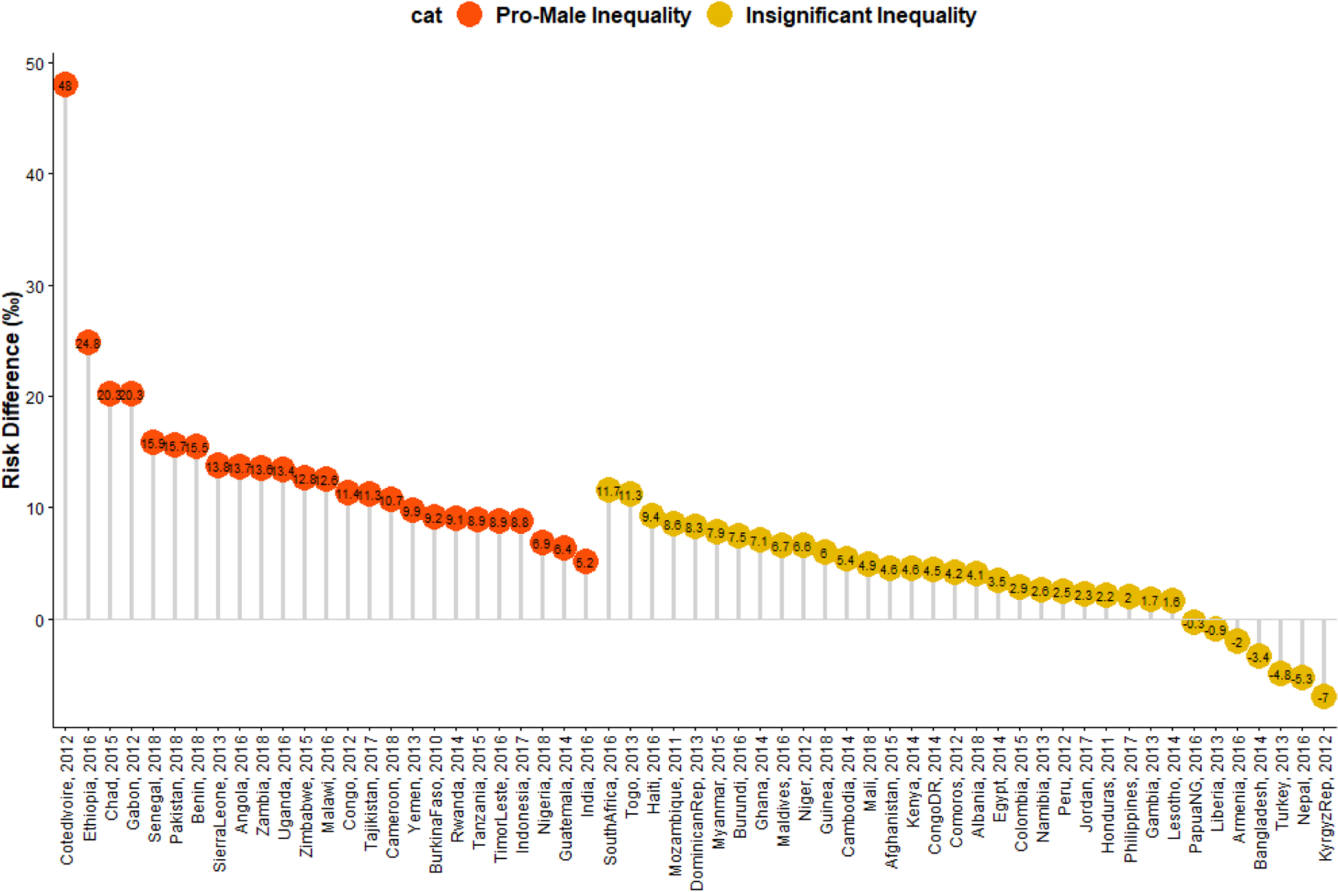
Risk difference between children from houses with sex differentials in under-five-deaths by countries in LMIC

**Fig. 3:**
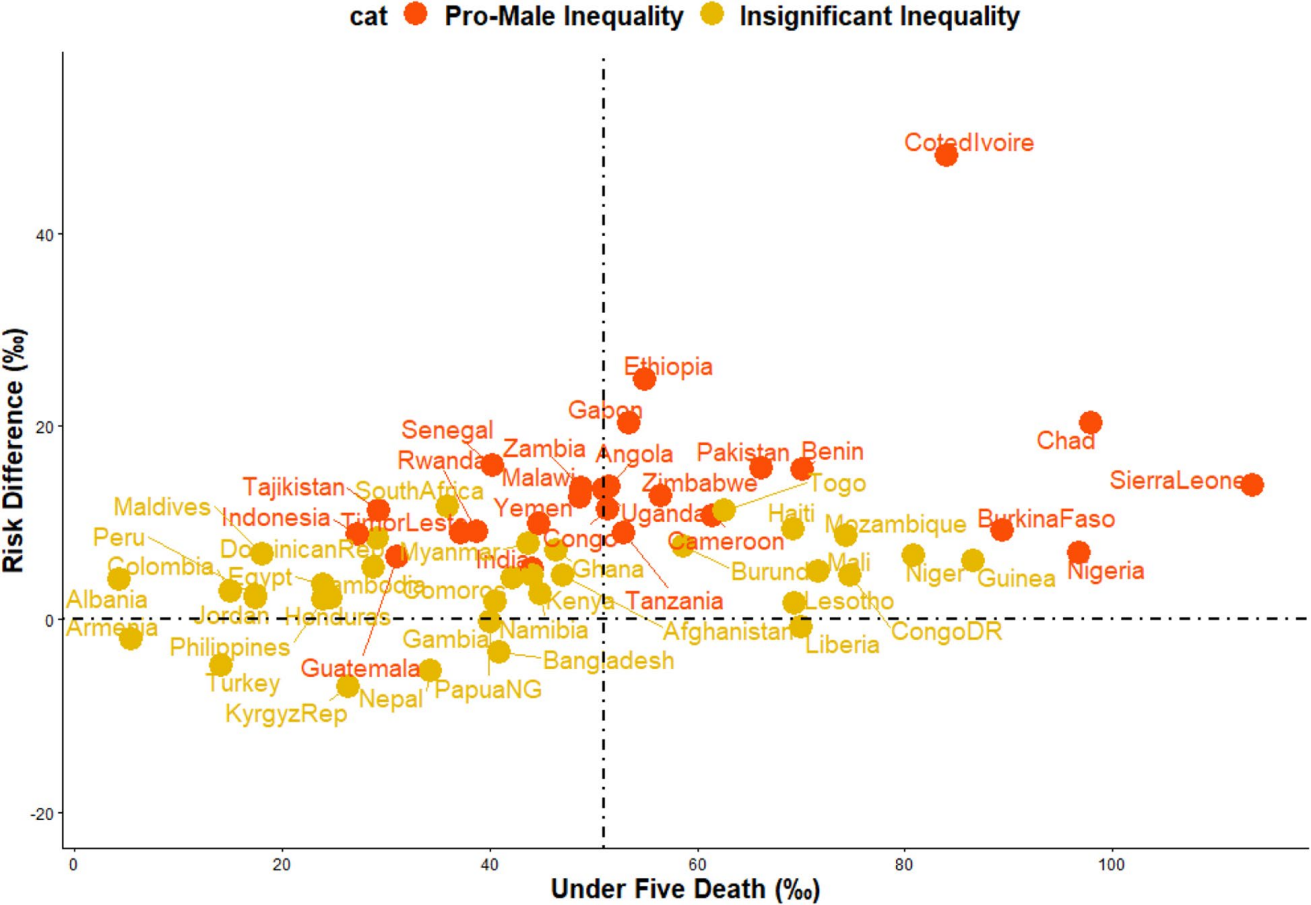
Scatter plot of rate of under-five-deaths and risk difference between sex of children in LMIC

We estimated both random and fixed effects of the RD. The fixed effects are the weighted country-specific RD and the random effects are the overall RD irrespective of a child’s country of residence as shown in Fig. 1. The purpose of the random effect was to estimate the overall prevalence and distributions of prevalence of U5MR among males and females irrespective of the countries the children are located. The fixed effects are to establish and identify the country-specific estimates. Charts were used to show the distributions of the RDs by the countries (Figs. 2 and 3). We categorized the countries into four distinct categories based on their prevalence of U5D and the size of their RD: (i) High U5D and high pro-male inequality (ii) High U5D and high pro-female inequality countries (iii) Low U5D and high pro-male inequality (iv) Low U5D and high pro-female inequality (Fig. 3). The Mantel-Haenszel (MH) Odds Ratio (OR) and tests of heterogeneity of ORs were used to ascertain that the countries were different with regards to the odds of U5D among the male and female children. A test of homogeneity of ORs among all the countries with a significant OR of U5D was also used to determine if the odds of having U5D in those countries were homogenous. Lastly, the multivariable-adjusted logistic regression method was applied to the pooled cross-sectional data from the U5D pro-male countries to carry out a decomposition analysis using the multivariable Fairlie decomposition analysis procedures. Sampling weights were applied in all our analyses to adjust for unequal cluster sizes, stratifications and to ensure that our findings adequately represent the target population.

Multicollinearity among the independent variables was tested using the “colin” command in Stata version 16. The command provided the Variance Inflation Factor (VIF). The VIF is approximate of 1/(1-*R*^*2*^) ranging from 1 to infinity. The *R*^2^-value is obtained by regressing the *j*^*th*^ independent variable on other independent variables. All variables with VIF>2.5 were removed from the regression analysis. Literature has shown concerns about VIF >2.5 [18]. Health insurance cover, media access, paternal employment status, type of cooking fuel and housing material were not captured in some countries and were dropped in the decomposition analysis. The decomposition analysis was conducted by obtaining the logit estimates of the magnitude of contributions of the factors to gaps in U5D between males and females as the dependent variable among those countries that had significant RDs.

#### Decomposition analysis

We applied multivariable Fairlie Decomposition Analysis (FDA) based on the binary regression model. The FDA is one of the decomposition techniques used in the quantification of the contributions to differences in the prediction of an outcome of interest between two groups in multivariate models [19]. The method is an extension of the Blinder-Oaxaca Decomposition Analysis [20–22], which has been roundly criticized for inefficiency in handling the logit and probit model [22, 23]. The FDA was purposively developed for non-linear regression models including the logit and probit models [24].

The FDA was carried out by calculating the difference between the predicted probability for one group (say Group A – male children) using the other group's (say Group B female children) regression coefficients and the predicted probability for male children using its regression coefficients [23]. The Fairlie decomposition technique works by constraining the predicted probability between 0 and 1.

Fairlie et al. showed that the decomposition for a nonlinear equation *Y* = *F*(*X*), can be expressed as:

1 $${\overline{\mathrm{Y}}}^A-{\overline{\mathrm{Y}}}^B=\overset{1^{st}}{\overbrace{\left[\sum_{i=1}^{N^A}\frac{F\left({X}_i^A{\hat{\beta}}^A\right)}{N^A}-\sum_{i=1}^{N^B}\frac{F\left({X}_i^B{\hat{\beta}}^A\right)}{N^B}\right]}}+\overset{2^{nd}\ }{\overbrace{\left[\sum_{i=1}^{N^B}\frac{F\left({X}_i^B{\hat{\beta}}^A\right)}{N^B}-\sum_{i=1}^{N^B}\frac{F\left({X}_i^B{\hat{\beta}}^B\right)}{N^B}\right]}}$$

Where *N*^*A*^ is the sample size for group *J* (25). In Eq. (1), $\overline{\mathrm{Y}}$ is not necessarily the same as $F\left(\overline{\mathrm{X}}\ \hat{\beta}\right)$, unlike in BODA where *F*(*X*_*i*_*β*) = *X*_*i*_*β*. The 1st term is the part of the gap in the binary outcome variable that is due to group differences in distributions of *X*, and the 2nd term is the part due to differences in the group processes determining levels of *Y*. The 2nd term also captures the portion of the binary outcome variable gap due to group differences in unmeasurable or unobserved endowments. In other words, the explained factors are those factors attributable to gender differences in individual observable characteristics and life circumstances while the unexplained factors are related to gender differences in the unobservable characteristics and life circumstances.

The estimation of the total contribution is the difference between the average values of the predicted probabilities. Using coefficient estimates from a logit regression model for a pooled sample, ${\hat{\beta}}^{\ast }$, the independent contribution of *X*_1_ and *X*_2_ to the group, the gap can be written as

2 $$\frac{1}{N^B}X\sum_{i=1}^{N^B}F\left({\hat{\alpha}}^{\ast }+{X}_{1i}^A{\hat{\beta}}_1^{\ast }+{X}_{2i}^A{\hat{\beta}}_2^{\ast}\right)-F\left({\hat{\alpha}}^{\ast }+{X}_{1i}^B{\hat{\beta}}_1^{\ast }+{X}_{2i}^A{\hat{\beta}}_2^{\ast}\right)$$

and

3 $$\frac{1}{N^B}X\sum_{i=1}^{N^B}F\left({\hat{\alpha}}^{\ast }+{X}_{1i}^B{\hat{\beta}}_1^{\ast }+{X}_{2i}^A{\hat{\beta}}_2^{\ast}\right)-F\left({\hat{\alpha}}^{\ast }+{X}_{1i}^B{\hat{\beta}}_1^{\ast }+{X}_{2i}^B{\hat{\beta}}_2^{\ast}\right)$$

respectively. The contribution of each variable to the gap is thus equal to the change in the average predicted probability from replacing the group *B* distribution with the group *A* distribution of that variable while holding the distributions of the other variable constant. Further numerical details have been reported [23–27]. We implemented the FDA in STATA 16 (StataCorp, College Station, Texas, United States of America) using the “Fairlie” command.

## Results

### Sample characteristics and analysis of inequality

Table 1 shows that the overall sex ratio of the children was 1.06 (51.4% male). There were more reported males than female children in the survey across all the countries except for Angola (49.8%), Congo (49.6%), Congo Democratic Republic (49.7%), Lesotho (49.5%), Namibia (48.8%) and Zimbabwe (49.1%). The highest proportion of male children were found in Armenia (53.3%), Papua New Guinea and the Philippines (52.5% each). The overall weighted prevalence of U5D was 51 per 1000 children, 55 among males and 48 among females (*p*<0.001). The prevalence of U5D among male children ranged from 4 per 1000 children in Armenia to 120 in Sierra Leone, while it ranged from 2 in Albania to 107 in Sierra Leone among female children. The z-test of equality of prevalence among male and female children was statistically significant (*p*<0.05) in Burundi, Ethiopia, Malawi, Rwanda, Tanzania, Uganda, Angola, Cameroon, Chad, Congo, Gabon, Zambia, Zimbabwe, Benin, Burkina Faso, Cote d’Ivoire, Nigeria, Senegal, Sierra Leone, Tajikistan, Bangladesh, India, Pakistan, and Yemen (*p*<0.001).

Table 2 shows that U5D was highest among multiple births compared with singletons (21.6% vs 5.1% among males and 18.0% vs 4.4% among females). The lowest death rates were in the neighborhoods with the least SES disadvantage with 3.5% among males and 3.0% among females. All the explanatory variables considered were significantly associated (*p*<0.05) with the U5D among all the children combined and by gender divides.

### Risk Differences in U5D among male and female children

The risk differences, a measure of inequality, in the risk of having U5D among male and female children across the countries studied were presented in Figs. 1, 2 and 3. Also, a meta-analysis of the prevalence of U5D among male and female children in each of the countries was carried out and presented the results in Fig. 1. The prevalence of U5D was generally higher in male children in all the countries except Liberia in West Africa; the Kyrgyz Republic in Central Asia; Bangladesh and Nepal in Southern Asia; Armenia in Western Asia; Turkey in Southern Europe and Papua New Guinea in Oceania. Pro-female inequality was however not significant in any of these countries. Irrespective of regions, the fixed effects of pro-male differences in U5D were widest in Cote d’Ivoire (48/1000 children) while the fixed effect of pro-female RD was widest for Turkey 5.3/1000). The random effects, that is the RD of U5D irrespective of country of residence per 1000 children was 7.5 (95% Confidence Interval (CI): 5.8-9.1), evidence of significant overall pro-male inequality in U5D. The greatest contribution (weight) to the random effect was found in India at 2.9% while the least was in Lesotho at 0.7% as shown in Fig. 1.

In Figs. 2 and 3, the red and orange colors indicate statistically significant pro-male inequality and insignificant inequality respectively. Based on RD, five of the nine countries in Eastern Africa, five of the six countries in Middle Africa, two in Southern Africa and four countries in West Africa showed statistically significant pro-male inequality. Three countries in Southern Asia, one country each in Central Asia, Western Asia, Central America and the Caribbean (Figs. 1, 2 and 3). The level of the heterogeneity of the RDs was 67% (*p*<0.01).

### Relationship between the prevalence of under-five deaths and magnitude of inequality

The relationships between the prevalence of U5D and the magnitude of male-female inequality, a function of RD, across the 59 countries involved in this study are presented in Fig. 3. Countries such as Cote D’Ivoire, Chad, Sierra Leone, Nigeria and Burkina Faso had high U5D and high pro-male inequality; Liberia and Lesotho had high U5D and high pro-female inequality, Tajikistan, South Africa, Senegal and Egypt low U5D and high pro-male inequality while countries such as Turkey and the Kyrgyz Republic had low U5D and high pro-female inequality.

### Decomposition of gender inequality in the prevalence of under-5 death

The Mantel-Haenszel pooled estimate of the odds ratio (OR) of having U5D controlling for the countries of the children. We estimated OR = 1.17 (95% CI: 1.14-1.19) and tested a null hypothesis: OR=1; and obtained z = 155.45 and *p* = 0.000 and (ii) Test of heterogeneity: *X*^*2*^ = 71.83, degree of freedom (d.f.) = 58, and *p* = 0.000, I-squared (variation in OR attributable to heterogeneity) = 19.3%. Of the 59 countries, statistically significant pro-male odds ratio (pro-male inequality) was found in only 25 countries. The countries were Afghanistan (*p*=0.001), Angola (*p*=0.000), Benin (*p*=0.001), Burkina Faso (*p*=0.022), Cambodia (*p*=0.026), Cameroon (*p*=0.048), Chad (*p*=0.000), Cote D’Ivoire (*p*=0.000), Ethiopia (*p*=0.000), Haiti (*p*=0.015), India (*p*=0.000), Indonesia (*p*=0.002), Kenya (*p*=0.018), Malawi (*p*=0.004), Mozambique (*p*=0.045), Niger (*p*=0.049), Nigeria (*p*=0.001), Rwanda (*p*=0.043), Senegal (*p*=0.038), Sierra Leone (*p*=0.002), Tajikistan (*p*=0.015), Tanzania (*p*=0.000), Timor Leste (*p*=0.019), Uganda (*p*=0.000), and Zambia (*p*=0.027).

We then computed Mantel-Haenszel pooled estimate of the odds ratio (OR) of having U5D among the children in the 25 countries with pro-male inequalities while controlling for the countries. We had OR= 1.18 (95% CI: 1.16-1.21) and tested the homogeneity ORs: *X*^*2*^ = 50.49, d.f. = 24, and *p* = 0.001.

**Fig. 4:**
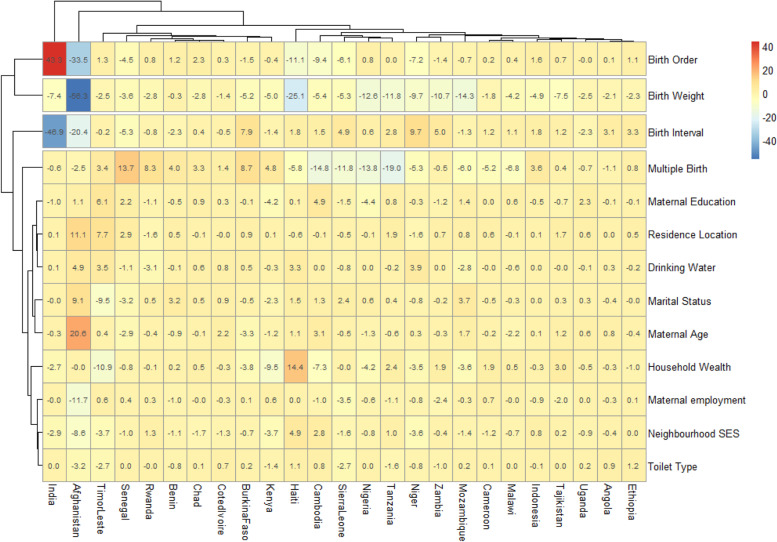
Decomposition of pro-male inequality attributable to compositional effects of under-five death determinants in 25 countries

These 25 LMIC pro-male inequality in U5D were included in the FDA. Figure 4 show the detailed decomposition of the part of the pro-male inequality caused by compositional effects of the determinants of U5D. The “explained” (compositional component) are depicted by red color while the “unexplained” (structural component) portions of the pro-male inequalities are depicted by the blue color in Fig. 4. The lighter the red color, the lower the percentage contribution of the “explained” portion and the lighter the blue color, the lower the percentage contribution of the “unexplained” portion. There were wide variations in the factors associated with the pro-male inequalities across the countries.

We found a connection (clustering) among birth order, birth weight, birth interval and having multiple births as factors associated with U5D, while other variables formed another cluster. Different factors had the largest association with U5D in different countries. In most countries, birth order, birth weight, birth interval and multiple births contributed most to U5D. Specifically in India, the largest contributions to pro-male inequality in U5D were birth interval (47.0%) and birth order (43.3%). In Afghanistan, birth weight, birth order and birth interval contributed 56.0%, 34.0% and 20.0% of the unexplained determinants of U5D respectively, while maternal age contributed 21.0% of the explained determinants. For Timor Leste, the greatest contributors to the inequalities were household wealth quintile, rural-urban differences in place of residence, and mothers’ marital status, while birth interval and birth weight were the greatest contributors to pro-male inequalities in Ethiopia. The contribution of the neighborhood factors to U5D inequality were mostly from explained determinants and they were not as strong as the contribution of individual factors as seen in Fig. 4. The highest contribution by residence location was seen in Afghanistan (11.1%) and Timor Leste (7.7%), while neighborhood socioeconomic class had even less contribution to U5D inequality with the highest contribution were seen in Haiti (4.9%) and Cambodia (2.8%).

## Discussion

In this study, we identified sexual inequality in the burden of U5D in LMIC using pooled data from 59 countries. Also, we quantified the individual- and neighborhood-level factors explaining the male-female differences in U5D. This study showed that U5D in most LMIC had a high-risk difference with pro-male inequality but with variations in the contributions of the determinants of U5D. However, most of the identified determinants were explainable. This showed that the natural female advantage at survival was still at play in many of these countries [28] and this reflects the level of development of the health system that is required to provide the platforms to reverse the natural male child’s predisposition to an early death. It also implies that the identified determinants can still be addressed to reduce U5D in these countries.

The four scenarios that considered the prevalence of U5D and the level of inequality provided thought-provoking insight into the spread and persistence of U5D in the countries studied. The natural scenario of high U5D and high pro-male inequality was expected in countries where the health system is underdeveloped [29, 30]. However, the explanation for this gender inequality in U5D may be misleading as subtle gender discrimination against female children is still seen in the affected countries. For example, for Nigeria, Adeyinka et al made predictions using under five mortality data from 1964 to 2017 that there will be a switch to pro-female inequality in the future [31]. This prediction was made based on the estimates from the use of artificial intelligence and modelling techniques to project the future pattern of U5D in Nigeria. Similar discrimination against the female children has been reported in India as well where male infants were being selectively vaccinated compared with females [32], giving the males a higher odd of survival. This is already evidenced in the higher number of U5D being recorded for female infants in some states in India [33]. Other discriminations against female infants which results in higher female mortality include selective abortion of female fetus, higher household fund dedicated for the care of male infants and general neglect of female children [33]. If the discrimination against female children is not effectively addressed [34], the gains from reduced U5D from improved health system will be subsumed sooner or later.

Also, the countries with high pro-male inequality (including Cote D’Ivoire, Chad, Sierra Leone, Nigeria and Burkina Faso) need to learn from countries with low U5D and make extra concerted efforts to reduce U5D with the already available strategies which are effective. Implementation strategies may need to be reviewed to identify problem areas that have been hindering the progress of halting U5D in these countries. Of the 10 countries with excess female mortality as reported by Alkema *et al* [35], only Bangladesh and Nepal still had pro-female inequality in this study. This suggests a reduction in female discrimination in the remaining eight countries (Afghanistan, Bahrain, China, Egypt, India, Iran, Jordan, and Pakistan) [35]. It is also interesting to observe that both India and China were long known to contribute significantly to pro-female inequality in Asia [10] but India in this study had pro-male inequality similar to reports from an earlier study [30]. This might be as a result of under-developed health system which still exist in some states in India [36]. Countries with high U5D and high pro-female inequality (like Liberia and Lesotho in this study) usually have the problem of female inequality as the U5D pattern does not fit into the natural pattern. High gender inequalities have been reported from both Liberia [37] and Lesotho [38] with the latter country also struggling with the effect of HIV which have been shown to have a higher impact on women [39].

Countries such as Turkey and the Kyrgyz Republic with low U5D and high pro-female inequality have some underground mechanisms that promote discrimination among females. This is because, with a reduction in overall U5D, the female survival advantage is expected to be preserved. For example, Turkey has been shown to have a culture of male preference and male children receive more attention than females [40]. Altindag et al. reported that women in Turkey preferentially use contraceptives following the birth of a male child which results in more spacing of children and give the male children a better opportunity to survive [40]. However, such use of contraceptives was not done following the delivery of a female child thereby giving them less chance of survival. There is also a high level of female inequality in the Kyrgyz Republic despite improvement in childhood anthropometric indices at the national level, even though poverty still contributes significantly to stunting [41].

Infection is still responsible for a good percentage of childhood illnesses in many LMIC countries because of suboptimal living standards and inadequate childhood vaccinations and male children are more at risk of death because of the less resistance to infection due to their genetic make-up [8]. The facilities and skills to support premature infants and infants with respiratory distress are also not readily available and the male child is also more predisposed to developing both conditions than their female counterparts [41, 42]. The countries with no significant sex disparity in U5D were mostly the upper-middle-income countries that have more resources to ensure better living conditions and provide improved health care services that can mitigate the peculiar health challenges that predispose the male child to higher mortality. This trend, however, suggests inequality as it points to likely female discrimination [43] because an improvement in the health system is expected to improve the survival of both male and female children. There is a need for further investigation to understand why there are no differences in the U5D in both sexes in these countries.

It is, however, important to note that there were countries that were not as economically buoyant as these earlier ones but still had no sex disparity in U5D. They include Tajikistan, Kyrgyz, Cambodia, the Philippines and Honduras. A closer examination of the focus and investment in the health sector of these countries provides some likely reasons for the absence of disparity. For example, in Tajikistan, there have been giant strides in economic growth in the last two decades which has resulted in a drastic reduction in poverty level among the populace because of investment by the World Bank. This includes investment in Under Five (U5) health [44] with significant improvement in under-five nutritional indices and healthcare workers’ management skills for childhood diseases [45]. However, as earlier pointed out, there is a need for further research to exclude discrimination against female children which may be responsible for the inequality in U5D seen in these countries.

The contributions of the explained (compositional) and unexplained (structural) components among countries with pro-male U5D inequality varied across the affected countries. In India, birth order conspicuously contributed to this inequality but there have been conflicting reports on  how birth order affects U5D in this country. While Singh and Tripathi reported that U5D was associated with birth order one and two from nationally representing data [46], Sahu et al reported that the risk for U5D was higher among birth order 4 and above in rural communities in India [47]. The argument in the first study was that lower birth order was associated with younger maternal age which could affect the skills required for under five care and predispose to U5D. Also, higher birth order in a rural community may stretch the already limited resources that are needed to ensure optimal care of children. The mothers in the second study were also mostly uneducated and this is a strong predictor of U5D. However, both birth order and birth weight were unexplained contributors to pro-male U5D in Afghanistan. In this country, it was reported in an earlier research that both large and small for gestational age and birth interval lesser than 24 months were associated with early neonatal deaths and the males significantly had a higher odds of dying than females [48]. These are in consonance with the findings from the current study. However, reports from another study in the same country showed that birth interval of 48 months and the male gender were protective from LBW, a condition that is among the leading cause of neonatal mortality [41]. This appears contradictory to the findings from this study as Afghanistan had a pro-male U5D inequality. The role of birth weight in the promotion of male inequality in Afghanistan need to be further investigated for better understanding. Multiple birth was also an explained contributor in Senegal but unexplained in Tanzania. The associated complications of multiple birth (like prematurity and perinatal asphyxia) have been well documented but its role in pro-male U5D inequality needs to be understood in order to address it in Tanzania. The last authors however reported that the male child was less likely to have LBW. Further investigation is required to ascertain the reason for this. In addition to the high pro-male inequality in U5D among these LMIC, there was high heterogeneity in the RD and this may be due to the different levels of health system development and variations in the predisposing factors as shown in the variation in the explained components of U5D. This is likely related to the differences in the stage of economic development and the priority given to U5 health care among these countries. Whereas it is logical to expect that countries with high U5D will have pro-male inequality.

All the independent variables included in our model were significantly associated with U5D just as reported in earlier literature [16, 49–52]. The result of the Fairlie decomposition analysis shows that U5D was due to more of explained components than the unexplained component which implies that reduction of U5D is possible if more strategic efforts are employed in LMIC income countries. Among the variables considered, maternal education appears to be an important determinant that is associated with U5D as it can influence the presence or otherwise of the other factors [12]. A woman who is educated is more likely not to have an under-age marriage [53, 54] so she will not have her babies too early and even if she has the children late, she is more likely to have supervised antenatal care and delivery [55, 56] that will ensure the safety of her baby. An educated woman is also likely to have access to the media, have health insurance [57] and be employed. Her partner is also more likely to be employed and even if she is unmarried, she will be in a better position to successfully head a household. It is worth reiterating that female-headed households were associated with less U5D in this study which is similar to what has been reported earlier in the literature [51, 58]. The likely reason is a better understanding of maternal and childhood conditions and this will lead to less bureaucracy in the decision-making process about accessing health care services for children.

Furthermore, an educated woman is more likely to have the socioeconomic advantage that will prevent her from being poor and have fewer factors that can predispose her to have low birth weight babies like malaria and HIV [12]. If she has a low birth weight baby, she will have better access to good management and not patronize substandard treatment centers. She is also more likely to do family planning which will help in spacing her babies and this will prevent her from having too many babies. Therefore, an investment in female education by these LMIC will go a long way in reducing U5D. For the few unexplained components of the determinants, more investigative researches are required to explore them. For example, it is important to understand why birthweight was an unexplained component of U5D in Afghanistan and why the contribution of birth interval to U5D cannot be explained in India.

The contribution of neighborhood determinants to U5D inequality was however not as much as those by individual factors. This may be as a result of shared cultures and national policies whose effects are likely to be far-reaching and this could have mitigated the differences expected in U5D as a result of the different categories of socioeconomic classes and locations of residence. Although residence location had the highest contribution to U5D inequality in this study in Afghanistan, there have been contrasting reports of how location affects U5D in this country. Kibria et al reported that rural infants were more likely to die in Afghanistan in their study of a nationally representative population of under-fives, but this association became insignificant after adjustment for maternal age and place of delivery [59]. This contrasts with reports from other countries [60–62] where residence in rural areas was associated with higher newborn mortalities, higher incidence of LBW and lower APGAR scores (both are leading factors for mortality).

### Study strengths and limitations

This study is one of the first analyses in LMIC that investigated individual and neighborhood factors contributing to sexual inequalities in U5D across 59 LMIC. The use of large nationally representative data enhanced the quality of our findings in terms of generalizability. Also, the FDA applied in our study is an improvement over the commonly used Blinder-Oaxaca decomposition analysis which has been reported to be inefficient in handling logit and probit models [21–23].

However, the study is not without some limitations. First, the measure of child mortality which is dependent on information provided by mothers may underestimate the actual rate as a result of recall bias. Most mothers may not be comfortable with talking about their dead children, and so, may not give accurate responses. The traditional practices in some countries also forbid parents from reporting the death of their children. Secondly, the cross-sectional nature of the study means that causal inferences cannot be made from our findings.

## Conclusions

In conclusion, significant sex inequality exists in U5D among LMIC with mostly male pro-inequality. There were different determinants for this male pro-inequality in the structural and compositional components considered across the different countries concerned. However, the pattern of this sex inequality reflected the presence of both weak health systems and female inequality. These countries will need to address the failing health system, address gender inequality and invest in female education to stem the tide of preventable U5D in LMIC if the SDG 3 is to be achieved.

### Contribution to Knowledge

The contribution of the study to knowledge is in two folds: (i) it identified countries with significant gender differences in under-five deaths. (ii) it quantified the contributions of the explored characteristics to the gaps/inequalities in sex differential in under-five deaths among countries with significant differences.

## Data Availability

The datasets supporting the conclusions of this article are available at http://dhsprogram.com.

## Abbreviations

CI: Confidence Interval
DHS: Demographic and Health Survey
FDA: Fairlie Decomposition Analysis
IRB: Institutional Review Board
LMIC: Low- And Middle-Income Countries
MDG: Millenium Development Goals
PSU: Primary Sample Unit
RD: Risk Difference
SES: Socioeconomic Status
SDG: Sustainable Development Goals
U5: Under-Five
U5C: Under-Five Children
U5D: Under-five deaths
UNICEF: United Nations International Children’s Emergency Fund
WHO: World Health Organization
